# Geophysical and multi-criteria decision methods for delineating groundwater potential in coastal terrains: a study from Port Sudan

**DOI:** 10.1038/s41598-026-35127-y

**Published:** 2026-01-21

**Authors:** Musaab A. A. Mohammed, Abazar M. A. Daoud, Mahmoud M. Kazem, Sarkhel H. Mohammed, Norbert P. Szabó, Péter Szűcs

**Affiliations:** 1 Faculty of Earth and Environmental Sciences and Engineering, University of Miskolc, Miskolc, 3515 Hungary; 2 College of Petroleum Geology and Minerals, University of Bahri, Khartoum, Sudan; 3 Faculty of Earth Sciences, Red Sea University Res Sea State, Port Sudan, Sudan; 4 Department of Mineralogy and Geology, University of Debrecen, Egyetem Tér 1, Debrecen, 4032 Hungary

**Keywords:** Port Sudan, Gravity data, Edge detection, Geophysical inversion, AHP, Groundwater recharge, Geophysics, Hydrogeology

## Abstract

Groundwater resources in arid, semi-arid, and coastal regions are of vital importance due to the scarcity or complete absence of reliable surface water sources. Port Sudan city is now serving as the administrative capital following the country’s political instability. As a result, the city has witnessed a massive influx of internally displaced people, placing pressure on its already fragile water resources. The region is underlain by Precambrian basement terrains, restricting groundwater occurrence to structurally controlled aquifers and alluvial deposits. This study integrates gravity data analysis with the analytical hierarchy process (AHP) to delineate potential groundwater zones in the area. Structural features were extracted from gravity data using edge detection techniques, including vertical and horizontal derivatives, tilt angle derivative, and analytical signal. A density map of the identified structures was generated and integrated with other groundwater recharge-controlling factors including geology, rainfall, land use, slope, and drainage density within an AHP framework. The multi-criteria evaluation resulted in a groundwater potential map delineating three distinct zones: low (41.5%), moderate (13%), and high potential (45.5%). These zones were validated using 2D gravity inverse modeling constrained by boreholes data along two profiles. This integrated approach provided a preliminary yet effective tool for groundwater exploration in complex basement terrains and supports decision-making for further detailed hydrogeological and geophysical investigations in Port Sudan and similar arid environments. Incorporating more detailed geophysical analyses could further enhance subsurface characterization and improve groundwater potential assessments.

## Introduction

Water scarcity continues to pose a significant global challenge, particularly in arid and semi-arid regions where the availability of freshwater resources is minimal and threatened by population growth and climate change impact^1^. In arid coastal regions dominated by crystalline basement terrains, groundwater often constitutes the most viable and sustainable source of freshwater for domestic and industrial use^2^. However, these terrains are typically characterized by complex and heterogeneous hydrogeological systems. Groundwater occurrence in such settings is discontinuous, spatially variable, and difficult to predict; therefore, investigating groundwater potential in these environments presents considerable challenges^3^. Traditional approaches such as hydrogeological mapping, exploratory drilling, and geophysical surveying, though valuable, are often labor-intensive and financially burdensome, particularly when applied over large and remote areas^4^. Accordingly, there is a growing imperative to develop accessible, scalable, and cost-effective methods for groundwater exploration in these terrains.

Geological structures play a fundamental role in groundwater occurrence and movement, particularly in basement terrains, where primary porosity is negligible and groundwater storage is predominantly confined to fractured zones and weathered layers^5,6^. Consequently, the identification and characterization of subsurface structures are essential steps in any hydrogeological investigation, especially in complex and water-stressed basement regions^7^. Generally, structural mapping has relied on remote sensing techniques, geological field mapping, and geophysical methods. Remote sensing, through satellite imagery has proven particularly useful for mapping surface lineaments and geological boundaries^8–11^. However, its capacity is inherently limited to features that manifest visibly at the surface. Many deep-seated structures that play a vital role in groundwater dynamics do not produce discernible surface expressions and thus remain undetected.

In this context, gravity data offers a non-invasive alternative that overcomes many of the limitations associated with remote sensing and conventional geological mapping^12^. Gravity surveying measures variations in the Earth’s gravitational field caused by subsurface density contrasts^13^. The use of gravity data for lineament detection and structural analysis has significantly advanced groundwater exploration, especially in regions where surface indicators are ambiguous or absent. Moreover, in addition to structural characterization, gravity data can also be used to infer lithological variations through gravity inversion techniques^14–16^. By converting gravity anomalies into subsurface density models, inversion allows for the delineation of different rock types and the estimation of their thicknesses^17^. This capacity to map both structural discontinuities and lithological heterogeneities makes gravity data a highly effective tool in the early stages of groundwater exploration, where resources for extensive drilling and surveying are limited.

Relying solely on structural information may lead to oversimplified interpretations and increased uncertainty in delineating groundwater potential zones^18^. Therefore, it is advisable to integrate additional groundwater-influencing parameters, to develop a more comprehensive and reliable assessment. Various multi-criteria decision-making approaches have been developed, allowing for the systematic weighting of contributing factors^19^. Among these, the analytical hierarchy process (AHP) has been widely and successfully applied due to its transparency, ease of implementation, and ability to incorporate expert judgment in the decision-making process^20,21^. AHP provides a structured framework for evaluating the relative importance of each parameter, enabling the integration of diverse groundwater-influencing factors into a single decision-support model^22^. Due to the inherent subjectivity involved in assigning weights, there is a potential for bias or inconsistency in the final outcomes. As a result, it is essential to validate the results of the AHP model against independent subsurface indicators to assess the reliability and effectiveness of the model. Most AHP-based studies validate their results using well hydraulic data^23,24^; however, this approach is often unreliable, as well hydraulic data are influenced by transient factors like seasonal recharge and pumping. These temporal data do not necessarily reflect sustainability of aquifer potential^25^. More robust validation should incorporate stable subsurface indicators which provide more reliable insights into aquifer structure and groundwater storage capacity.

Port Sudan city has recently become the de facto administrative capital following the country’s political instability. As a result, the city has witnessed a massive influx of internally displaced people, placing pressure on its already fragile water resources^26^. With limited precipitation and increasing urban water demands driven by population growth, the need for effective groundwater resource development has become more urgent than ever. Local communities in Port Sudan are facing acute freshwater shortages, with many households forced to rely on expensive desalinated water or irregular deliveries from tanker trucks which are options that are neither sustainable nor accessible to all population^27^. This deteriorating water security situation has escalated into a public health crisis and hindered economic development^28^. While several studies have broadly assessed groundwater potential in some parts of the Red Sea Hills, few have focused specifically on the Port Sudan area. These few studies relied on generalized geological mapping or remote sensing without detailed geophysical integration^29,30^. This research aims to develop an integrated and cost-effective approach for identifying potential groundwater zones using gravity and multi-criteria decision analysis. The goal is to enhance the understanding of subsurface hydrogeological conditions and support sustainable groundwater development in Port Sudan and similarly affected arid regions. Unlike many multidisciplinary studies that rely primarily on limited surface indicators and temporally variable subsurface validation methods, this study adopts a more stable and robust validation approach, grounded in geophysical data and supported by borehole-constrained inverse modeling. This cross-validation enhances the reliability and accuracy of groundwater potential zone delineation, making the method robust for use in complex basement terrains. It strengthens the credibility of the results and supports the creation of a more sustainable and practical groundwater potential map for informed decision-making.

## Study area

**Fig. 1:**
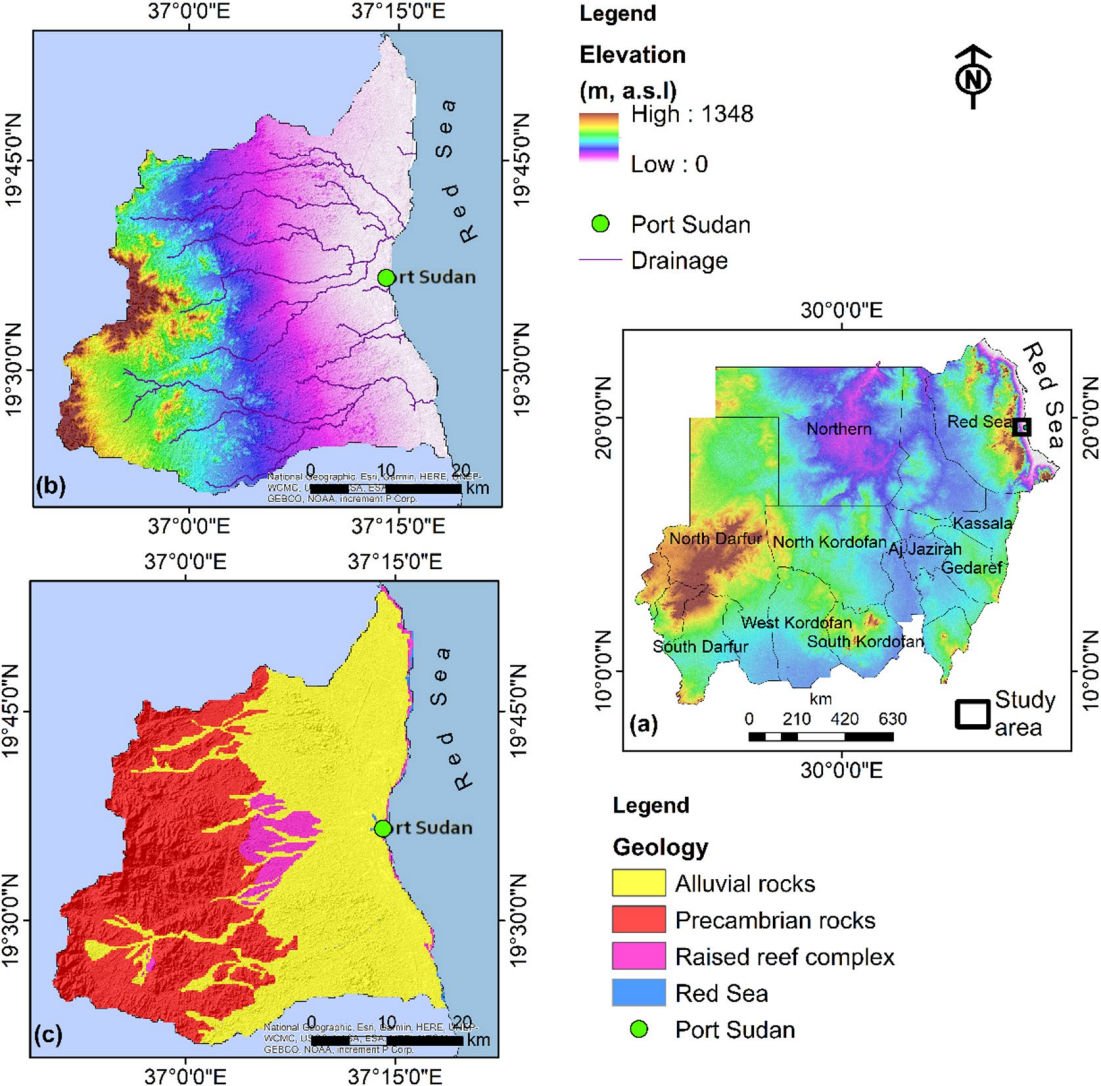
(**a**) The geographical location of the study area, (**b**) the digital elevation model of the study area, and (**c**) geology of the study area mapped within the ArcGIS (V. 10.8)^36^.

Port Sudan, positioned along Sudan’s eastern Red Sea coastline at approximately 19°35′ N latitude and 37°13′ E longitude (Fig. 1a). This area features diverse topography transitioning from a narrow coastal plain through foothills into the rugged Red Sea Hills, which rise over 1300 m to the west (Fig. 1b)^31^. The drainage system consists primarily of seasonal wadies flowing eastward from the highlands toward the coast. The climate is characterized as arid to semi-arid with minimal annual precipitation averaging at 200 mm, occurring mainly during winter months (November-March). Despite limited rainfall, the coastal location maintains relatively high humidity levels (60–80%), while extreme evaporation rates exceeding 1000 mm annually create a substantial moisture deficit^30^.

Port Sudan is situated within the Nubian Shield, part of the larger Arabian-Nubian Shield that formed during the Neoproterozoic Pan-African orogeny approximately 900 − 550 ma^32^. Eastern Sudan’s geological evolution is dominated by the opening of the Red Sea rift, which began approximately 30 ma when the Arabian Plate began separating from the African Plate. The Port Sudan area features several main geological units (Fig. 1c), beginning with the Precambrian Basement Complex composed primarily of metamorphic and igneous rocks including gneisses, schists, and granites^33^. Overlying these basement rocks are Mesozoic-Cenozoic sedimentary sequences, particularly along the coastal areas^34^. These include sandstones and shales from continental environments, limestones and evaporites from shallow marine environments, and coral reef deposits along the modern coastline. The youngest geological units consist of Quaternary deposits including alluvial and fluvial fans composed of consolidated sand, silt, and gravel^35^. The region exhibits multiple structural features related to its tectonic history, including NW-SE trending fault systems parallel to the Red Sea rift, horst and graben structures formed by extensional tectonics.

Groundwater is hosted in several aquifer systems. The Quaternary alluvial aquifers represent shallow, unconfined systems located primarily within wadi deposits and coastal alluvium. These aquifers are composed of sand, gravel, and silt, resulting in relatively high permeability and good potential for water extraction. However, their shallow nature makes them highly vulnerable to seawater intrusion in coastal zones^37^. The general direction of groundwater flow in alluvial aquifer follows the surface drainage pattern, moving from west to east toward the sea, in alignment with the topographic gradient^38^. Beneath these alluvial aquifer lies the Pre-Cambrian basement complex. Basement rock aquifers are characterized by low productivity, with groundwater primarily occurring in fractures and shallow weathered zones, where permeability has been secondarily enhanced^30^. These aquifer zones typically range in thickness from 5 to 20 m, although thicker sections may occur locally. Due to their limited porosity and storage capacity, these fractured and weathered aquifers hold only small quantities of groundwater. Water quality within these aquifers ranges from fresh to brackish, and recharge is highly variable, primarily influenced by rainfall and surface runoff events^39^.

## Materials and methods

**Fig. 2:**
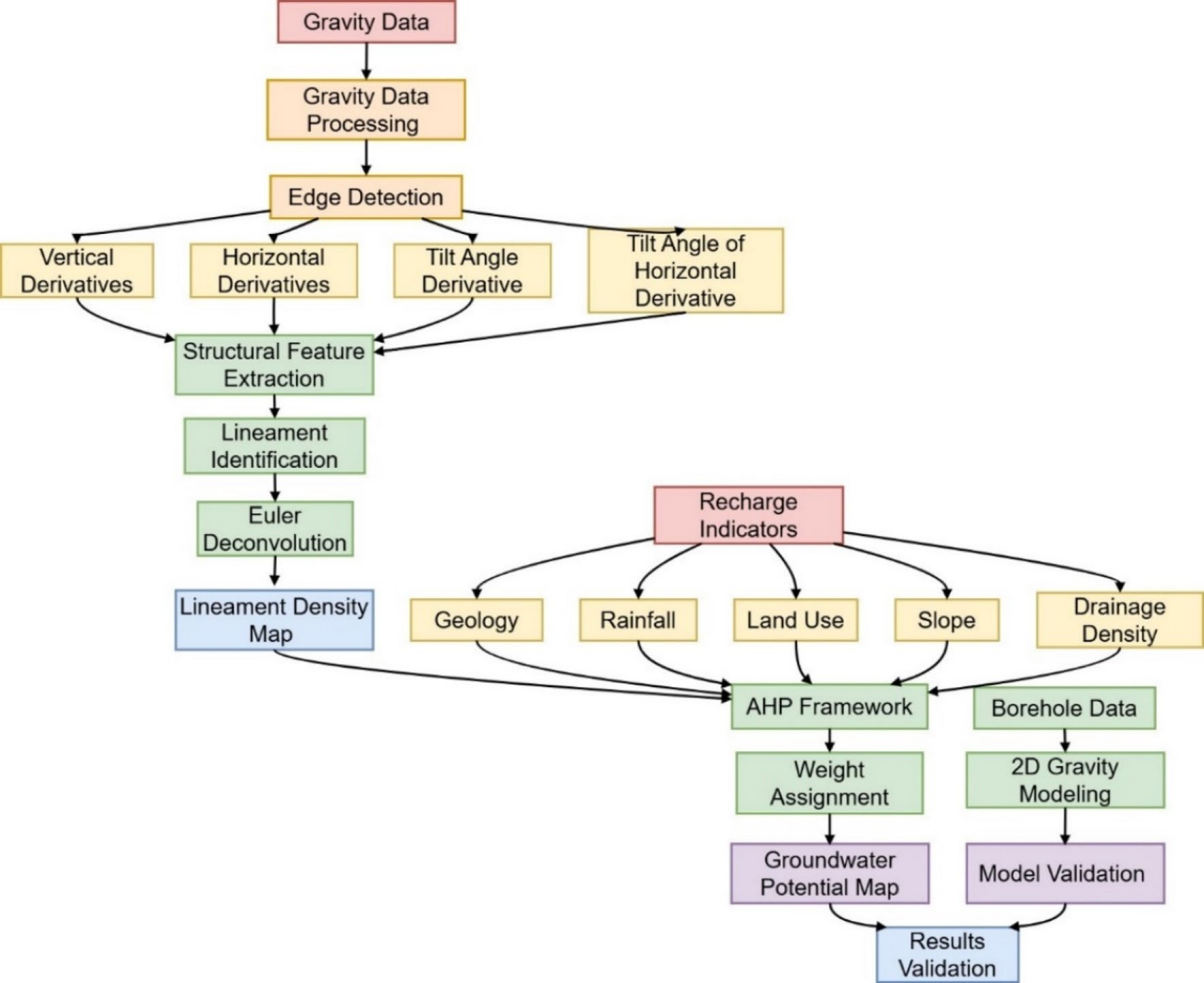
The workflow of the current study.

The workflow of this study combines gravity data interpretation with a multi-criteria decision-making approach to identify potential groundwater zones in the Port Sudan city (Fig. 2). First, gravity data were processed using edge detection techniques including vertical and horizontal derivatives, tilt angle and tilt angle of the horizontal derivative to map subsurface structural features. The depth of key lineaments was estimated using Euler deconvolution. A lineament density map was then produced and integrated with other thematic layers influencing groundwater recharge, including geology, rainfall, land use, slope, and drainage density. These factors were weighted and ranked using AHP to generate a groundwater potential map. The results were validated through 2D inverse modeling of gravity data and available borehole information.

### Gravity data

High-resolution satellite gravity data were obtained from the Global Gravity Model Plus (GGMPlus), which integrates multiple data sources including GOCE/GRACE satellite observations (10,000–100 km), EGM2008 (100–10 km), and topographic gravity information derived from SRTM topography^40^. The GGMPlus dataset provides gravity data at a spatial resolution of 7.2 arc-seconds (~ 200 m), enabling detailed mapping of short-wavelength gravity variations associated with near-surface geological structures. The global free-air gravity anomalies from GGMPlus range between − 456 mGal and + 714 mGal, with an overall accuracy of about ± 5 mGal globally and ± 15–25 mGal regionally across Africa, where ground-based validation is sparse^40^.

To derive the Bouguer anomaly, the SRTM2gravity model was employed, which uses 90 m Shuttle Radar Topography Mission (SRTM) elevation data to estimate the gravitational attraction of topographic masses^41^. A reduction density of 2.67 g/cm³, representing the mean crustal density of Precambrian basement terrains, was applied for the Bouguer and terrain corrections. This density value was tested against nearby geological data and previous geophysical investigations in the Red Sea Hills and provided the most stable anomaly field without introducing artificial regional trends. The gravitational attraction of topography was subtracted from the free-air gravity anomalies to obtain the complete Bouguer anomaly map, which forms the basis for subsequent edge-detection filtering and structural interpretation.

#### Anomaly separation and edge detection

The complete Bouguer anomaly map was analyzed using multiple potential field techniques to delineate subsurface geological structures. The regional–residual separation was performed using a Gaussian high-pass filter, with the cutoff wavelength determined objectively from power spectrum analysis^42^ of the Bouguer anomaly data in the wavenumber domain via Fast Fourier Transform (FFT)^43^. In this approach, the radially averaged logarithmic power spectrum was plotted against the wavenumber (k). Distinct linear segments on the log–power spectrum correspond to anomaly sources at different depths. The intersection (break point) between the regional and residual slopes indicates the transition frequency separating deep-seated and shallow sources. The corresponding wavelength at this point was used as the cutoff wavelength to design the Gaussian filter. This deterministic procedure minimizes subjectivity and ensures that the regional field represents deep crustal trends, while the residual component highlights short-wavelength anomalies associated with shallow structures and fault-controlled aquifers relevant to groundwater occurrence^44^.

Following anomaly separation, edge detection techniques were applied to the residual gravity anomaly map to delineate structural transitions. The first vertical derivative (FVD) of the gravity field (g) was calculated (Eq. 1) to enhance short-wavelength anomalies and suppress long-wavelength regional trends, thereby emphasizing shallow structural discontinuities. FVD is particularly effective in highlighting linear features with respect to vertical direction (z)^45^. Lineaments in the FVD output are typically identified along the zero-contour lines, which represent the inflection points in the gravity gradient^46^. The total horizontal gradient (HG) was computed (Eq. 2) to detect the horizontal rate of change in the gravity field^47^. The HG incorporates the gravity derivatives in the x (east-west) and y (north-south) directions. This method is sensitive to abrupt lateral changes in density, making it useful for locating vertical or steeply dipping geological boundaries. In this context, lineaments are identified along the zones of maximum amplitude in the horizontal gradient.

The tilt angle derivative (TAD) (Eq. 3) was used to normalize the gradient field by expressing the vertical derivative relative to the horizontal gradient^48^. TAD is particularly effective for enhancing both shallow and deep sources and for highlighting weak or subtle structures. Lineaments are typically interpreted along the zero-angle contours in the TAD map, which correspond to source edges. The tilt angle of the horizontal gradient (TAHG) applies the tilt function directly to the horizontal gradient (Eq. 4), enhancing source edge detection by balancing the response across varying source depths^49^. Lineaments in the TAHG map are generally extracted from amplitude maxima, providing a clear visualization of both dominant and subordinate geological boundaries^50^.

1 $$\:\mathrm{F}\mathrm{V}\mathrm{D}=\frac{\partial\:\mathrm{g}}{\partial\:\mathrm{z}}$$

2 $$\:\mathrm{H}\mathrm{G}=\sqrt{{\left(\frac{\partial\:\mathrm{g}}{\partial\:\mathrm{x}}\right)}^{2}+{\left(\frac{\partial\:\mathrm{g}}{\partial\:\mathrm{y}}\right)}^{2}}$$

3 $$\:\mathrm{T}\mathrm{A}\mathrm{D}={\mathrm{t}\mathrm{a}\mathrm{n}}^{-1}\left(\frac{\frac{\partial\:\mathrm{g}}{\partial\:\mathrm{z}}}{\sqrt{{\left(\frac{\partial\:\mathrm{g}}{\partial\:\mathrm{x}}\right)}^{2}+{\left(\frac{\partial\:\mathrm{g}}{\partial\:\mathrm{y}}\right)}^{2}}}\right)$$

4 $$\:\mathrm{T}\mathrm{A}\mathrm{H}\mathrm{D}=\:{\mathrm{t}\mathrm{a}\mathrm{n}}^{-1}\left(\frac{\frac{\partial\:\mathrm{H}\mathrm{G}}{\partial\:\mathrm{z}}}{\sqrt{{\left(\frac{\partial\:\mathrm{H}\mathrm{G}}{\partial\:\mathrm{x}}\right)}^{2}+{\left(\frac{\partial\:\mathrm{H}\mathrm{G}}{\partial\:\mathrm{y}}\right)}^{2}}}\right)$$

To estimate the depth of subsurface causative bodies and extract three-dimensional structural information, Euler deconvolution was applied to the gravity dataset. This technique utilizes the principles of Euler’s homogeneity equation to relate spatial gradients of the gravity field to the location and depth of density contrast sources^51^. The method operates by moving a window across the gravity data and solving for the source location and depth within each window, thus generating a spatial distribution of structural solutions. Euler deconvolution is based on Eq. 5 as

5 $$\:\left(\mathrm{x}-{\mathrm{x}}_{0}\right)\:\frac{\partial\:\mathrm{g}}{\partial\:\mathrm{x}}+\left(\mathrm{y}-{\mathrm{y}}_{0}\right)\:\frac{\partial\:\mathrm{g}}{\partial\:\mathrm{y}}+\left(\mathrm{z}-{\mathrm{z}}_{0}\right)\:\frac{\partial\:\mathrm{g}}{\partial\:\mathrm{z}}=-\mathrm{N}(\mathrm{g}-{\mathrm{g}}_{0})$$

where (x_0_​,y_0_​,z_0_​) are the coordinates of the unknown source location, (x, y,z) are the coordinates of the observation point, g_0_ is the regional background field which is assumed to be constant and determined through power spectrum analysis of the Bouguer anomaly data, N is the structural index (SI), which defines the rate of fall-off of the gravity field and is related to the geometry of the source. Different SIs are used depending on the assumed geometry of the source. For example: SI = 0 for contacts or faults, SI = 1 for dikes or sills, SI = 2 for cylindrical bodies. In this study, SI of 0 was selected because it corresponds to contacts or step-like geological features, which are dominant in the study area due to the presence of sharp lithological boundaries and fault-controlled contacts between different lithological units^29^.

#### Inversion of gravity data

A two-dimensional interpretation of gravity data was conducted to delineate the structural configuration and basement topography within the study area. The gravity modeling and inversion were carried out using the GM-SYS module in Geosoft Oasis Montaj (v. 8.4), which relies on the forward modeling approach originally introduced by Talwani^52^ and incorporates inversion techniques adapted from^53^. The forward modeling process begins with the construction of a conceptual subsurface model composed of multiple layers, each defined by a series of connected vertices forming polygonal shapes. These polygons simulate geological formations with assigned density values, enabling the depiction of vertical variations in subsurface properties^16^. The forward model for the polygon approximation is expressed using Eq. 6 as

6 $$\:\varDelta\:\mathrm{g}=2G\varDelta\:\rho\:\sum\:_{i}\left[{x}_{i\:}\mathrm{ln}\left(\frac{{r}_{i+1}}{{r}_{i}}\right)+{z}_{i}\left({\theta\:}_{i+1}-{\theta\:}_{i}\right)\right]$$

where Δg is the gravity anomaly (in mGal), which is the difference between the observed and theoretical gravity, *G* is the universal gravitational constant = 6.674 × 10^− 11^ m^3^ kg^− 1^ s^− 2^, Δρ is the density contrast between the anomalous body and surrounding material (in kg/m³), x_i_ and z_i_ are the coordinates of the i^th^ vertex of the polygon, r_i_ is the distance from the observation point to vertex i, and θ_i_ is the angle made by the line connecting the observation point and the i^th^ vertex, with respect to a horizontal axis,

The inversion process iteratively adjusts the model geometry and density values to minimize discrepancies between observed and simulated gravity anomalies^54^. This misfit is quantitatively assessed using the root mean square (RMS) error, which evaluates the difference between measured and computed gravity data. In this study, an RMS threshold of ≤ 5 mGal was considered acceptable, aligning with standards commonly applied in basement terrain investigations to ensure a geologically meaningful fit. To ensure geological realism and improve model stability, the inversion incorporates physical constraints derived from existing geological data, including borehole data. Two boreholes were used to constrain the gravity inversion modeling. These wells reach a depth of approximately 35 m and provide basic lithological descriptions used to validate the subsurface layering inferred from the gravity data. The optimization of model parameters is guided by a least-squares approach, using the Marquardt (1963) algorithm to iteratively refine the solution and achieve a best-fit model based on the geophysical data and known subsurface conditions.

### Analytical hierarchy process (AHP)

AHP is a multi-parameters remote sensing and GIS-based analytical method used to map the groundwater potential zones through a comparative analysis of the groundwater conditioning factors^56^. The AHP calculations were performed using a customized Microsoft Excel sheet developed by^57^, which automates pairwise comparison, consistency ratio calculation, and weight normalization. This study used six factors, including geology, slope, land use/land cover (LULC), drainage density (DD), and lineament density (LD). The AHP involves two levels of application, which are arranging the parameters in a hierarchical form and comparing them pair wisely. It permits the quantification of the relative importance of each parameter in the groundwater potential assessment. These parameters are given ranks and weights that depict their importance in groundwater potential zoning. The ranking and normalized weight range between 1 and 9^58^. The higher rank is assigned to the factor or subfactor that has the higher impact on the groundwater potential zoning. In this study, the ranking and the weights are assigned to each factor based on the researcher’s knowledge about the groundwater conditions in the study area.

The normalized weights derived from the comparison matrix (Table 1) are verified using the consistency ratio of the matrix (CR) based on the consistency index (CI) and the random consistency index (RCI) using Eq. 7^59^. The CI, in return, is calculated using Eq. 8. The CR measures the logical consistency of the pairwise comparisons used to generate the AHP weight matrix. A CR value below 0.1 (10%) is generally considered acceptable, indicating that the judgments are sufficiently consistent for reliable decision-making.

7 $$\:\mathrm{C}\mathrm{R}=\frac{\mathrm{C}\mathrm{I}}{\mathrm{R}\mathrm{C}\mathrm{I}}$$

8 $$\:\mathrm{C}\mathrm{I}=\frac{{\uplambda\:}\mathrm{m}\mathrm{a}\mathrm{x}-\mathrm{n}}{\mathrm{n}-1}$$

Where $\:\lambda\:max$ is the principal eigenvalue and n is the number of the conditioning factors.

The conditioning factors defined previously into sub-factors were scaled and weighted based on their impact on groundwater availability using a comparison matrix for each sublayer. The groundwater potential zones (GWPZs) are then calculated using Eq. 9 as

9 $$\:GWPZ=\:\sum\:_{i=1}^{n}\sum\:_{j=1}^{m}{W}_{i}\times\:\:{CV}_{j}$$

where W_i_ represents the normalized weights for each conditioning factor, CV_j_ is the weights of the sub-factors (normalized feature), n is the factor number, and m is the number of subclasses for each factor.

**Table 1:** Pairwise comparison between different groundwater influencing factors.

	Geo	LULC	DD	LD	Slope	Rainfall
Geo	1	6	4	3	4	2
LULC	1/6	1	1/3	1/4	1/3	1/5
DD	1/4	3	1	1/3	2	1/3
LD	1/3	4	3	1	3	1/2
Slope	1/4	3	1/2	1/3	1	1/3
Rainfall	1/2	5	3	2	3	1

*Geo = Geology

## Results and discussion

### Lineaments extraction

**Fig. 3:**
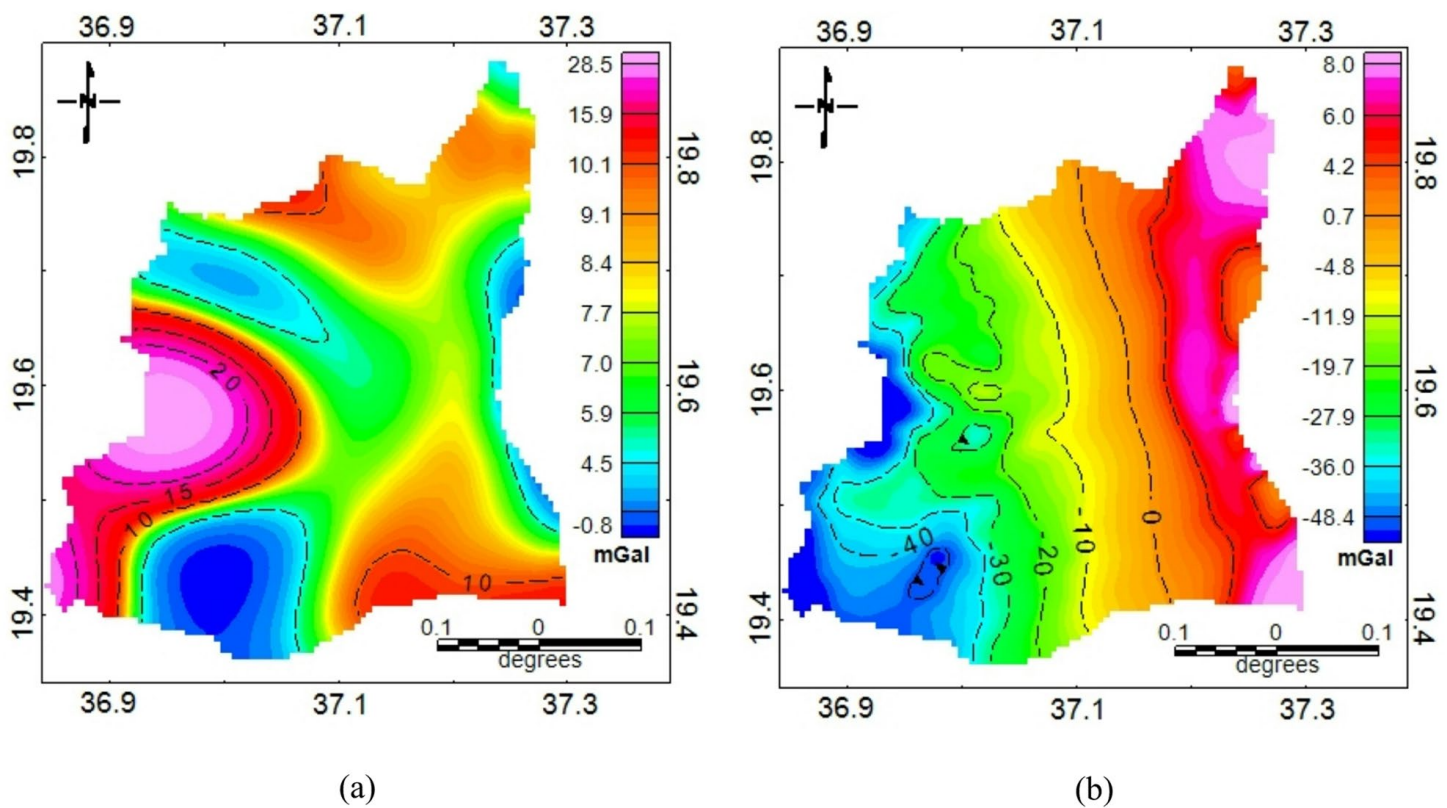
(**a**) The free air anomaly map and (**b**) complete Bouguer anomaly map.

**Fig. 4:**
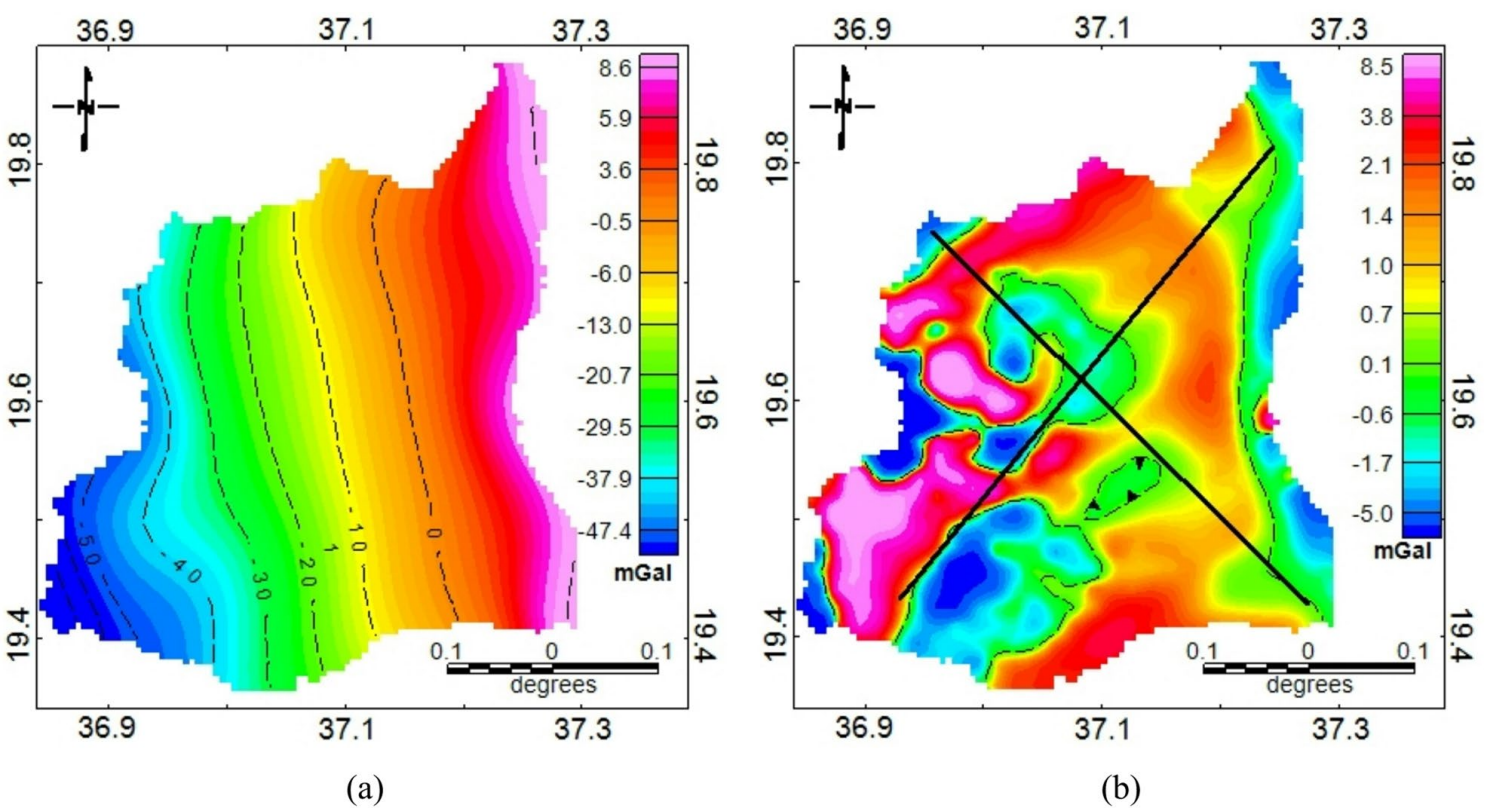
Anomaly separation results showing (**a**) regional anomaly and (**b**) residual anomaly.

The gravity anomaly maps (Fig. 3) provide the geophysical basis for subsurface interpretation in the study area. The free-air anomaly map (Fig. 3a) highlights broad regional variations influenced by elevation and deep crustal features, while the Bouguer anomaly map (Fig. 3b) reflects subsurface density contrasts by correcting for topographic and terrain effects^60^. A clear east-west gradient in the Bouguer anomaly is indicated with anomalies ranging from approximately − 48.4 mGal in the west to around 8 mGal in the east. The gravity anomaly separation of Bouguer anomaly map revealed a clear distinction between the regional and residual components (Fig. 4). The separation using spectral analysis involves subjectivity in selecting the cutoff wavelength for the Gaussian filter. Its interpretation depends on familiarity with the geological context. To enhance confidence in the chosen regional field, the filtered results were cross-checked against known regional geological trends. Accordingly, the regional anomaly map (Fig. 4a) exhibits a smooth, long-wavelength trend, with gravity values generally increasing from west to east. This gradient is interpreted as the expression of deep-seated geological structures, possibly reflecting variations in crustal thickness and the depth to the crystalline basement.

In contrast, the residual anomaly map (Fig. 4b) highlights short-wavelength variations that are indicative of shallow subsurface features. These residuals reveal localized gravity highs and lows that are associated with density contrasts near the surface, potentially caused by faults, fractures, or variations in lithology such as weathered basement rocks and sediment-filled structural depressions. The residual map presents sharp gradients and localized anomalies that often align in linear or curvilinear patterns, suggesting the presence of buried faults or fracture zones. These features are of particular interest in groundwater exploration, especially in basement terrains where aquifer potential is often structurally controlled. The separation process successfully enhances the high-frequency signals that are critical for identifying shallow geological structures relevant to hydrogeological assessments.

**Fig. 5:**
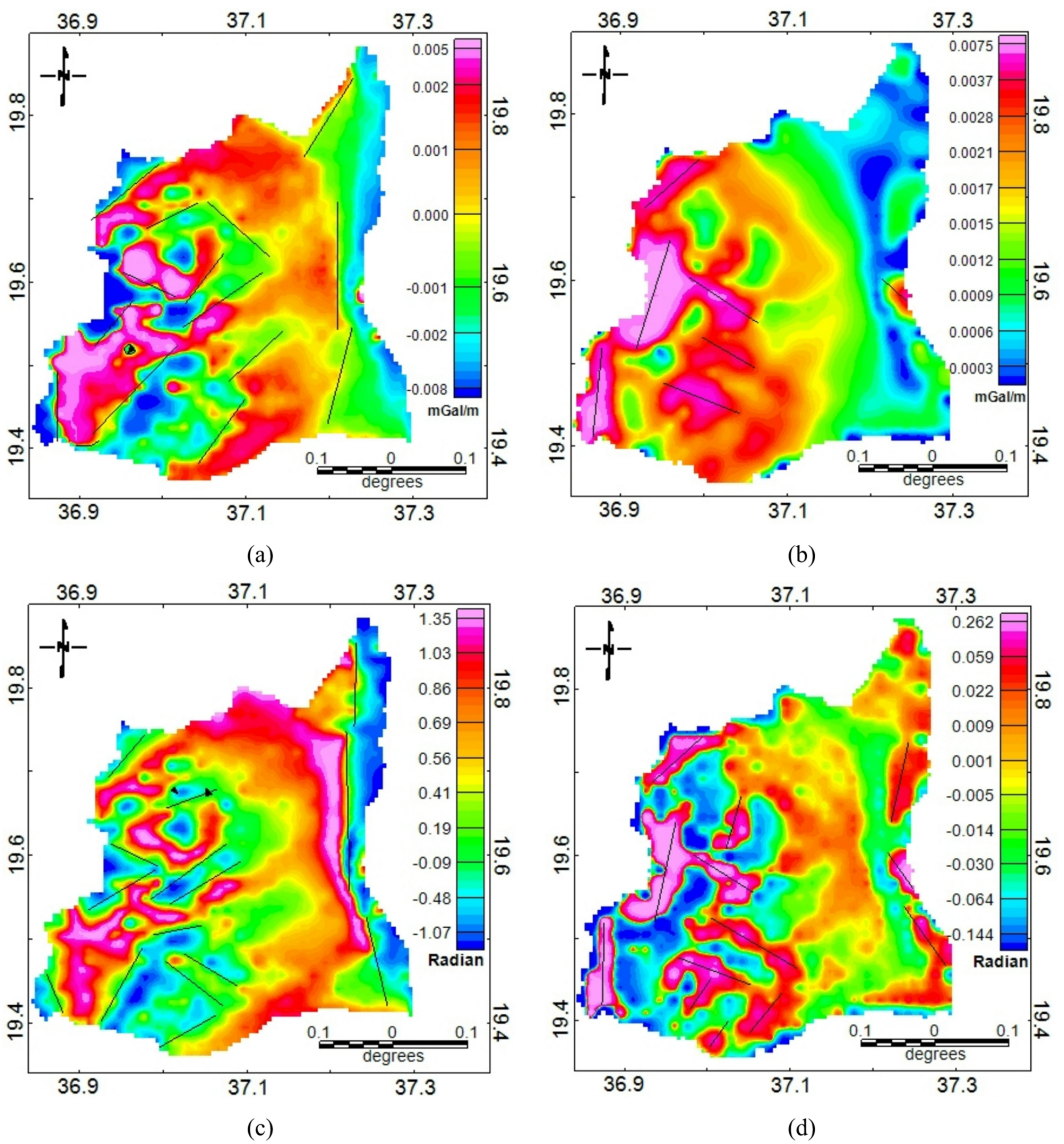
The lineaments extraction using (**a**) first vertical derivative, (**b**) horizontal gradient (**c**) tilt angle derivative and (**d**) tilt angle of horizontal derivative.

The structural interpretation was conducted using edge detection filters applied to the residual gravity anomaly map, including the FVD, HG, TDR, and TAHG. Each filter enhances specific characteristics of the gravity field to delineate lineaments and geological boundaries. The FVD map (Fig. 5a) emphasizes high-frequency components and enhances shallow features, revealing several prominent linear structures with NE–SW and NW–SE orientations. The HGM filter (Fig. 5b), which is sensitive to lateral density contrasts, complements the FVD results by outlining sharp boundaries corresponding to geological contacts and faults. These zones of high gradient reflect abrupt changes in subsurface density and are interpreted as major structural discontinuities. The TAD map (Fig. 5c) enhances subtle features that may be obscured in other filters, helping to refine the structural interpretation, particularly in regions with low-relief anomalies. The TDR results indicate the presence of secondary fracture zones trending in a north-south direction. The TAHG (Fig. 5d) revealed a dense network of intersecting lineaments, particularly in the central and southeastern parts of the study area. The combined application of gravity-based filters provides a robust framework for structural interpretation and confirms that the dominant tectonic trends in the study area are NE–SW and NW–SE, with subordinate N–S trending shear fractures.

**Fig. 6:**
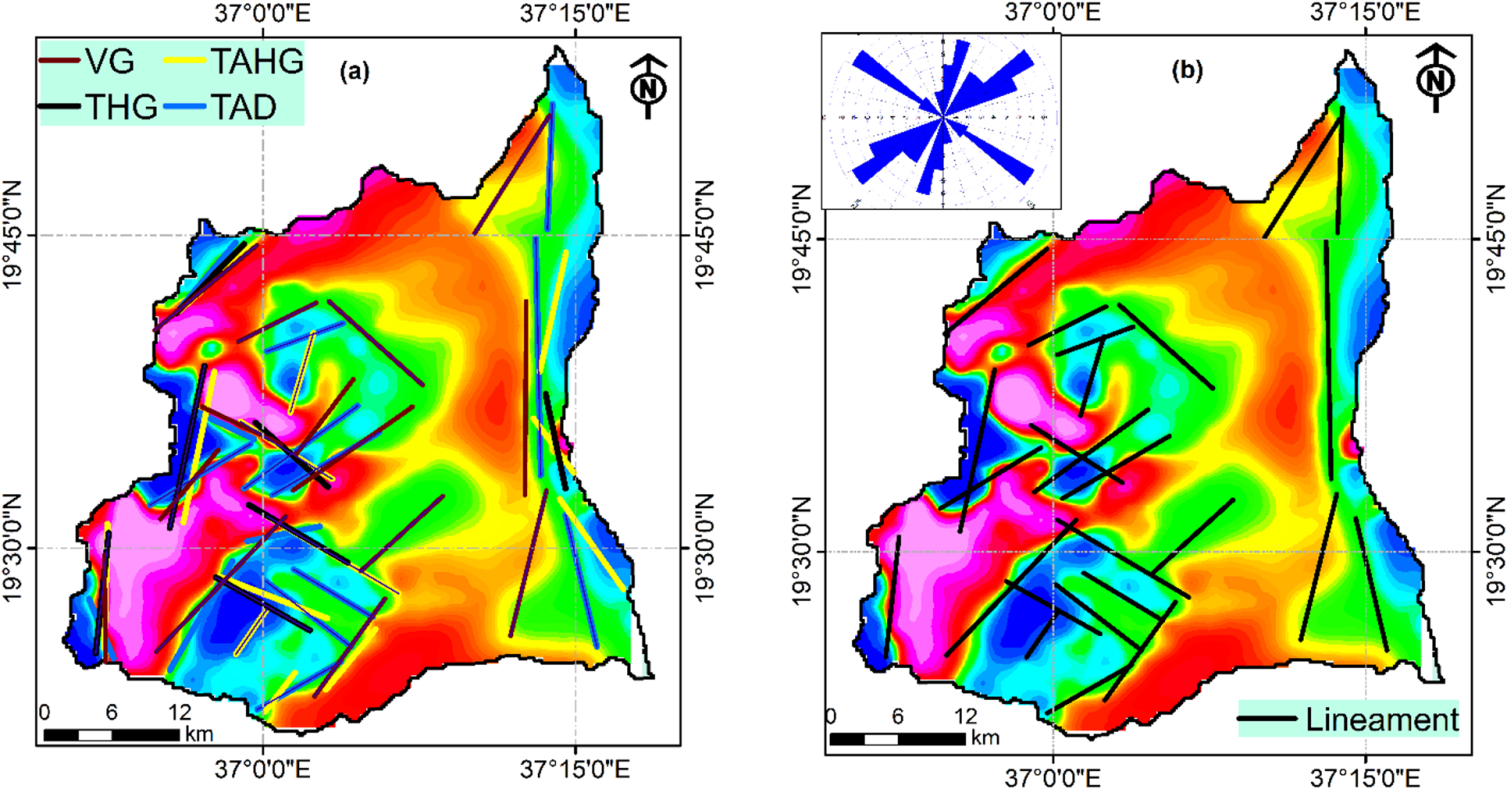
**(a)** Compilation of the lineaments extracted using different edge detection techniques and (**b**) filtered lineaments and their orientations.

The comparative analysis between FVD, HG, TAD, and TAHG methods successfully delineated subsurface discontinuities throughout the study area (Fig. 6a). Each method contributed complementary information to the integrated lineament map (Fig. 6b), enhancing the reliability of structural interpretation. The final integrated lineament map, produced through careful manual filtering of the combined results, demonstrates a pronounced dominance of NE-SW and NW-SE trending features, as visualized in the rose diagram (Fig. 6b). This pattern represents conjugate fracture systems associated with the regional tectonic stresses of the Red Sea rifting process, where NE-SW lineaments parallel the coastline orientation and NW-SE lineaments represent transform zones. This is consistent with regional structural observations where NE- to NNE-trending brittle faults and shear zones dominate the Red Sea hills^32,61^.

**Fig. 7:**
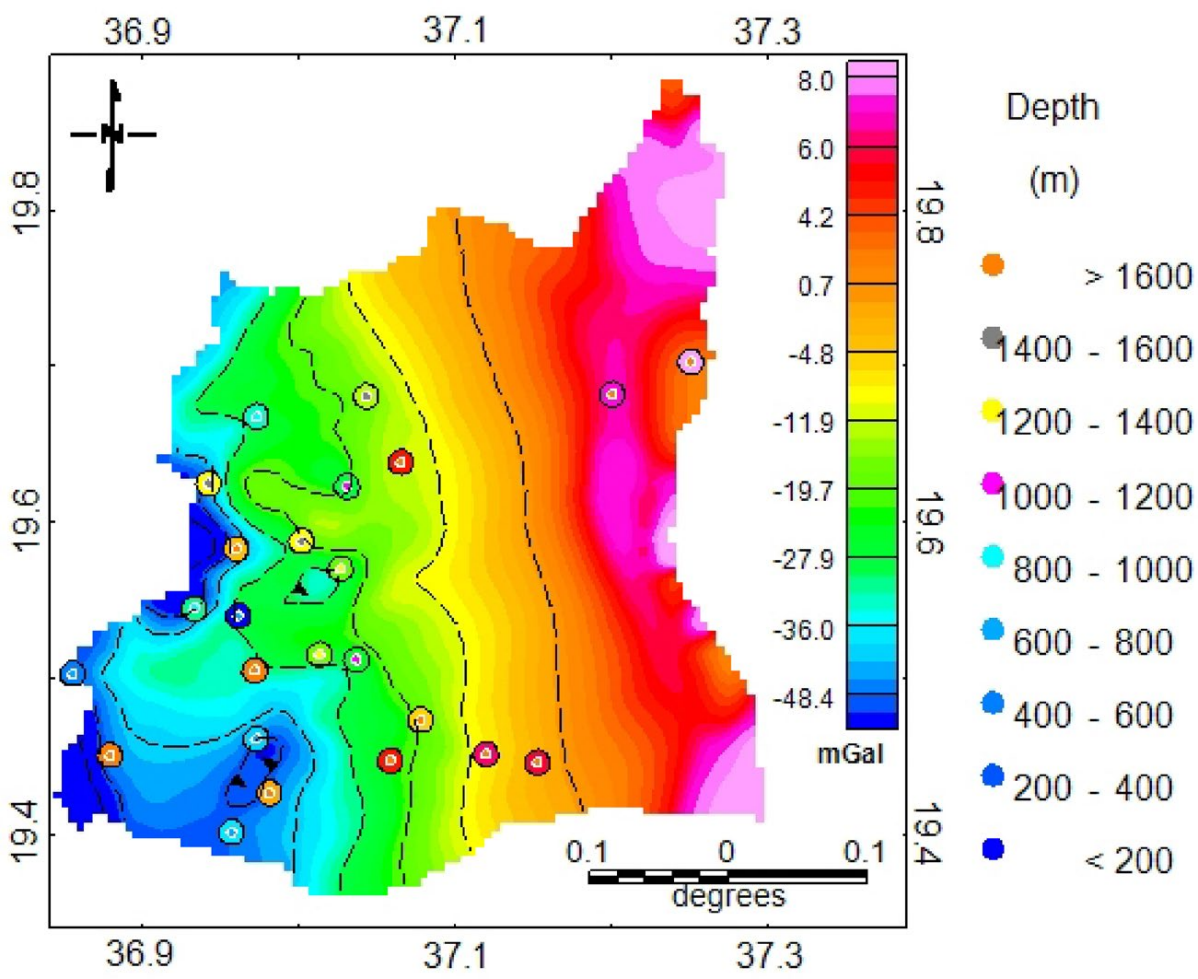
The depth of the delineated linemants using Euler deconvolution.

The central and western portions of the study area exhibit higher lineament density, suggesting zones of increased fracturing, while the eastern coastal region displays fewer but more continuous lineaments possibly indicating major fault zones. These zones also coincide with areas of higher structural complexity in the ANS, which have been shown to influence surface drainage patterns and subsurface groundwater behavior^5,62^. However, according to the depth estimation derived from Euler deconvolution of the identified linear features, the majority of these lineaments are located at depths greater than 50 m (Fig. 7). This suggests that they lie below the alluvial deposits and represent structural features within the fractured basement rock. The southwestern portion exhibits significantly deeper lineaments indicating depths exceeding 1600 m. The western sections of the study area are characterized by predominantly shallow lineaments representing depths of 200–800 m, with some features extending to less than 200 m. Shallower lineaments represent fracture systems with more immediate influence on near-surface and may serve as primary recharge zones and conduits for groundwater movement. Conversely, the eastern coastal zone, while characterized by fewer lineaments, shows more continuous and linear features confirming deeply rooted fault systems associated with major tectonic boundaries^63,64^. The deeper lineaments may indicate zones where groundwater can be transmitted over larger volumes, potentially sustaining wells that tap into fractured bedrock aquifers. However, their contribution to immediate recharge is usually less direct compared to shallow fractures.

### Recharge influencing factors

The AHP was employed to quantify the relative importance of various parameters influencing groundwater potential in the Port Sudan area, as summarized in Table 2. This systematic multi-criteria decision analysis approach enabled the objective weighting of six primary hydrogeological parameters and their respective sub-parameters based on their influence on groundwater occurrence and movement. These derived weights were subsequently applied to the corresponding spatial datasets to generate a composite groundwater potential index, thereby transforming qualitative hydrogeological understanding into a quantitative spatial assessment framework.

**Table 2:** Parameters and sub-parameters with their normalized weights used for groundwater potential zoning.

Parameter	Sub-parameter	Normalized sub-parameter weight (%)	Normalized parameter weight (%)
Geology	Alluvial deposits	60	37.1
	Coral reef	10	
	Basement rocks	25	
	Sea	5	
Slope [degree]	Low (0–5.59)	48	7.6
	Medium (5.6–12.69)	29	
	High (12.7–22.39)	15	
	Very high (22.4–54.8)	8	
LULC	Sea water	13	4
	Trees	73	
	Residential area	14	
Rainfall [mm]	Low (63.9–73.5)	40	24
	High (73.6–84.5)	60	
Drainage density [km/km^2^]	Low (0–0.239)	54	9.6
	Medium (0.24–0.47)	30	
	High (0.48–0.71)	16	
Lineament density [km/km^2^]	Low (0–1.6)	25	17.6
	Medium (1.61–3.3)	35	
	High (3.31–4.8)	40	

#### Geology

**Fig. 8:**
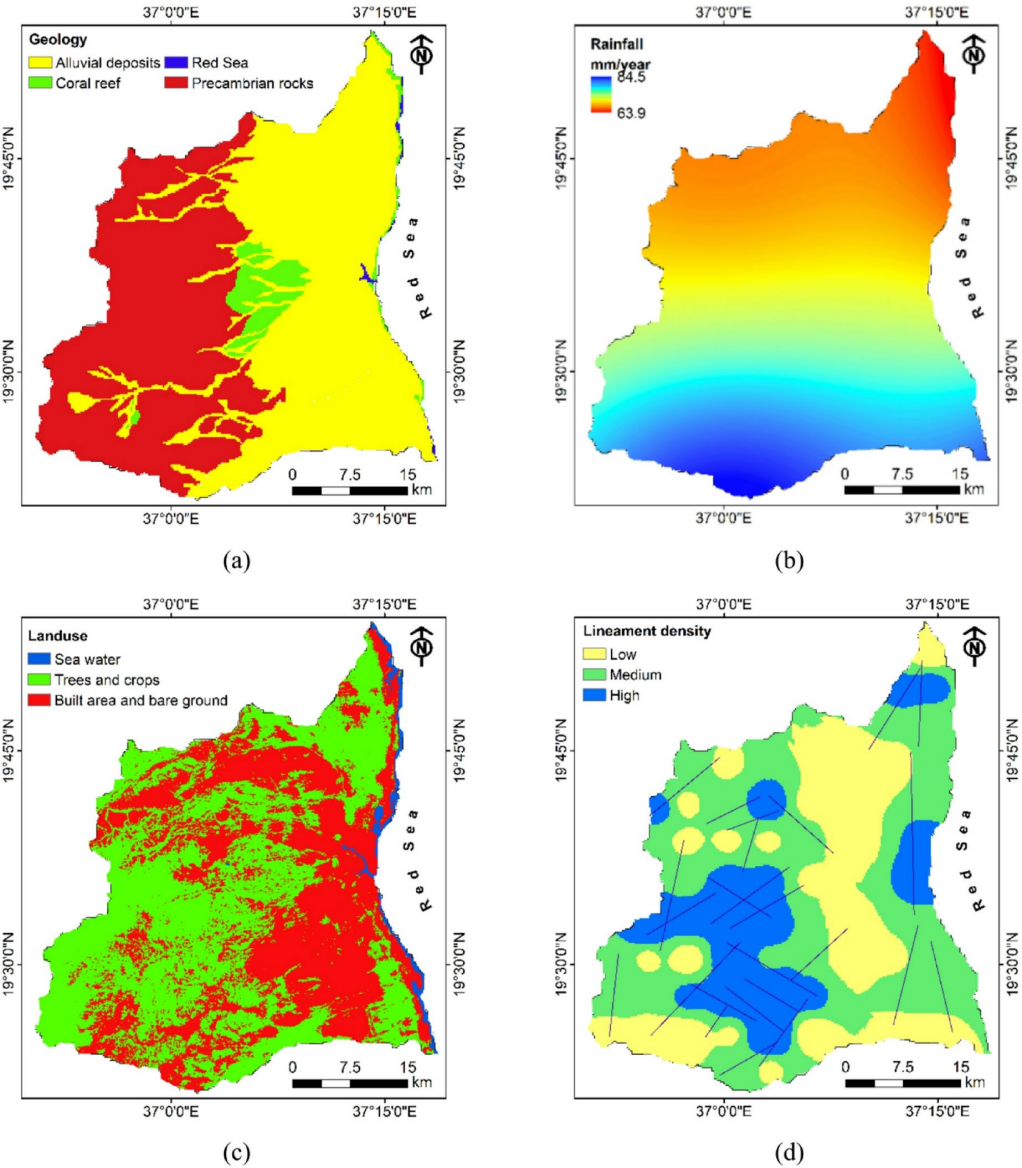

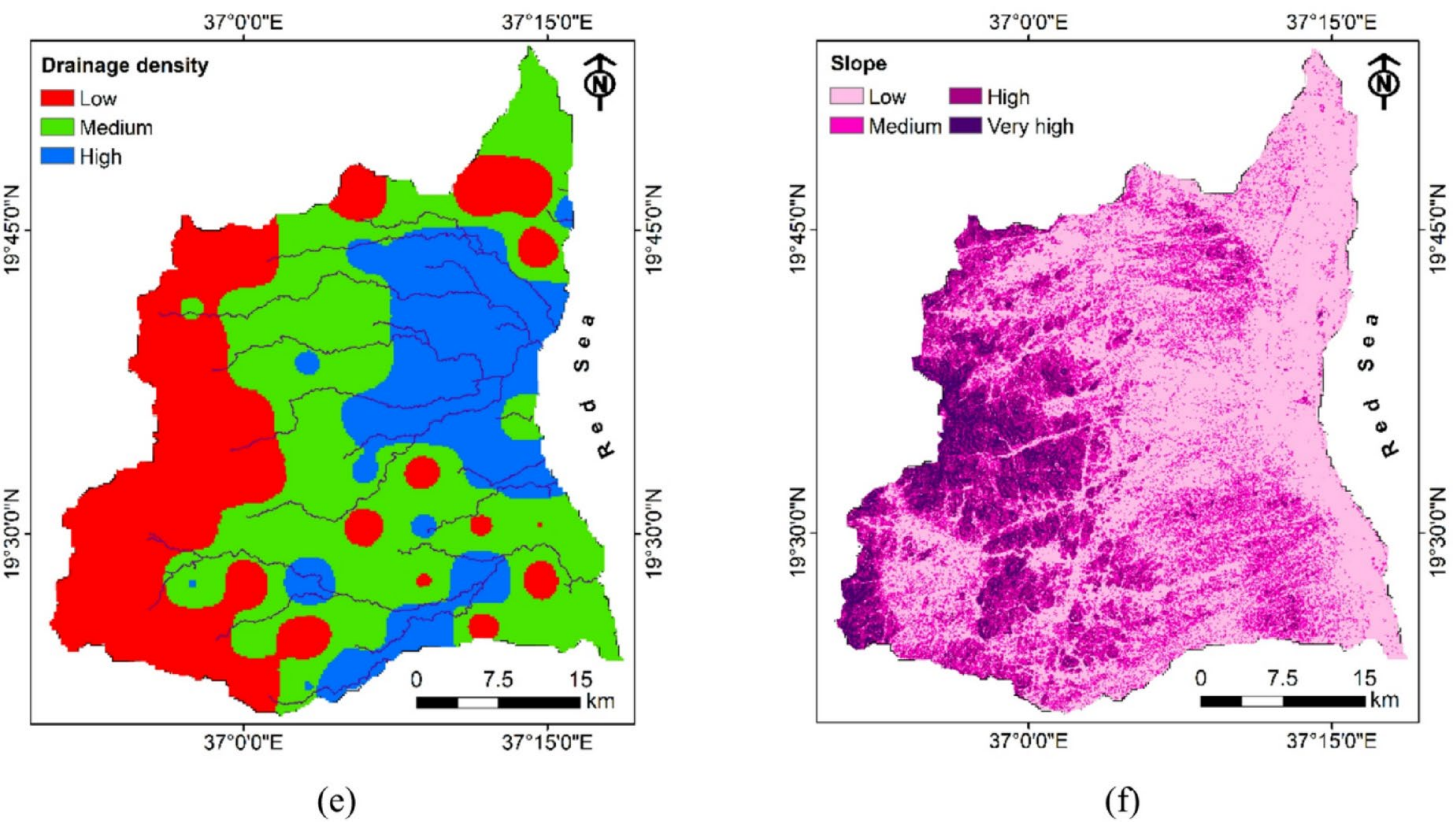
Groundwater recharge influencing factors including (**a**) geology, (**b**) rainfall, (**c**) landuse landcover, (**d**) lineament density, (**e**) drainage density, and (**f**) slope.

Geology emerged as the most influential factor with the highest parameter weight (37.1%), underscoring its fundamental control on aquifer characteristics and groundwater dynamics. Within this parameter, alluvial deposits received the highest sub-parameter weighting (60%) due to their superior hydrogeological properties - high primary porosity, excellent permeability, substantial thickness, and exceptional water-bearing capacity^30^. As illustrated in Fig. 8a, these deposits predominantly occupy the eastern portions of the study area, forming the most promising aquifer systems. Basement rocks, despite their limited primary porosity, received moderate weighting (25%) acknowledging their significant potential for secondary porosity through weathering and fracturing processes, particularly in areas where lineament intersections create enhanced hydraulic conductivity. These crystalline formations dominate the western regions of Port Sudan, providing groundwater primarily through fracture networks. Coral reef formations received considerably lower weighting (10%) due to their limited areal extent along the coastal fringe and variable permeability characteristics, while sea areas received minimal weighting (5%) as they represent non-potable water bodies unsuitable for freshwater extraction despite their hydraulic influence on coastal aquifers through saltwater interfaces.

#### Rainfall

Rainfall distribution, weighted at 24% overall, constitutes the third most significant parameter affecting groundwater recharge dynamics. The spatial variability in precipitation directly influences infiltration volumes and aquifer replenishment rates. The high rainfall category (73.6–84.5 mm) received considerably greater weight (60%) compared to lower rainfall areas (63.9–73.5 mm, weighed at 40%), reflecting the direct proportionality between precipitation intensity and recharge potential. This weighting acknowledges that even modest increases in rainfall within this semi-arid environment can significantly enhance groundwater recharge potential through greater soil moisture surplus. Figure 8b demonstrates a clear north-south rainfall gradient across Port Sudan, with higher precipitation in the northern regions gradually decreasing southward, suggesting enhanced recharge potential in the northern portions of the study area where infiltration opportunities are maximized under higher rainfall conditions.

#### Land use

Land use/land cover (LULC) was assigned the lowest parameter weight (4%) but provides crucial insights into surface permeability conditions and anthropogenic modifications affecting recharge processes. Tree-covered areas received remarkably high sub-parameter weight (73%) due to their capacity to enhance soil structure through organic matter addition, reduce runoff velocity through interception, increase surface roughness, and promote deep infiltration through root channel systems. Residential areas received substantially lower weighting (14%) due to increased impervious surfaces that reduce infiltration capacity and accelerate runoff^65^, while sea water areas received the lowest weight (13%) due to their non-contribution to freshwater recharge despite hydrological connectivity with coastal aquifers. Figure 8c illustrates that tree coverage (green areas) is extensive throughout the central and southern regions, providing favorable infiltration conditions despite anthropogenic modifications in residential zones (red areas) predominantly located in the central eastern portion of the study area.

#### Lineament density

Lineament density ranked as the second most influential parameter (17.6%), highlighting the critical importance of structural controls on groundwater movement in this tectonically active region influenced by Red Sea rifting. Higher lineament density zones (3.31–4.8 km/km²) received the greatest sub-parameter weight (40%) as these zones represent intensely fractured systems offering enhanced secondary porosity, permeability, and interconnectivity between aquifer units, facilitating groundwater storage and transmission. Medium density areas (1.61–3.3 km/km²) received substantial weighting (35%) reflecting their moderate contribution to groundwater potential through interconnected fracture networks, while low density zones (0–1.6 km/km²) received the lowest weighting (25%) due to their limited structural permeability. The lineament density layer assigned equal weight to all extracted lineaments, regardless of their depth. While this approach simplifies the integration process, it may overlook important differences in hydrogeological significance between shallow and deep structural features. Figure 8d reveals that high lineament density zones are concentrated primarily in the southwestern and eastern portions of the study area, coinciding with the previously identified intersections of major NE-SW and NW-SE trending structural features that form potential groundwater conduits.

#### Drainage density

Drainage density received a moderate parameter weight of 9.6%, functioning as an important indicator of surface water-groundwater interactions. Lower density areas (0–0.239 km/km²) received the highest sub-parameter weight (54%) compared to medium density zones (0.24–0.47 km/km², weighted at 30%) and high-density areas (0.48–0.71 km/km², weighted at 16%). This seemingly counterintuitive weighting acknowledges that while drainage networks reflect surface runoff pathways, areas with lower drainage density often indicate greater subsurface infiltration capacity, reduced surface runoff, and higher permeability conditions, thereby enhancing groundwater recharge potential^66^. Conversely, regions with high drainage density typically signify impermeable subsurface conditions that promote surface runoff rather than infiltration. Figure 8e illustrates that low drainage density areas are predominantly distributed in the central and southeastern regions, suggesting favorable infiltration conditions in these zones where surface water has greater opportunity to percolate into aquifer systems.

#### Slope

Slope emerged as a moderately influential factor (7.6% weight), significantly affecting infiltration rates and runoff-infiltration ratios. Gentler slopes (0–5.59.59°) received substantially higher sub-parameter weight (48%) compared to medium (5.6–12.69.6.69°, weighted at 29%), high (12.7–22.39.7.39°, weighted at 15%), and very high (22.4–54.8°, weighted at 8%) slope categories. This progressive reduction in weights with increasing slope angle reflects the inverse relationship between slope steepness and infiltration opportunity time, as gentler slopes facilitate greater water retention, reduced runoff velocity, and prolonged soil-water contact time that enhances percolation and recharge processes. The slope distribution map (Fig. 8f) reveals that the eastern coastal plains and portions of the central region exhibit predominantly gentle slopes highly favorable for groundwater recharge, while the western highlands feature increasingly steeper terrain that limits infiltration potential through accelerated runoff and reduced residence time of surface water.

### Groundwater potential zones (GWPZ)

**Fig. 9:**
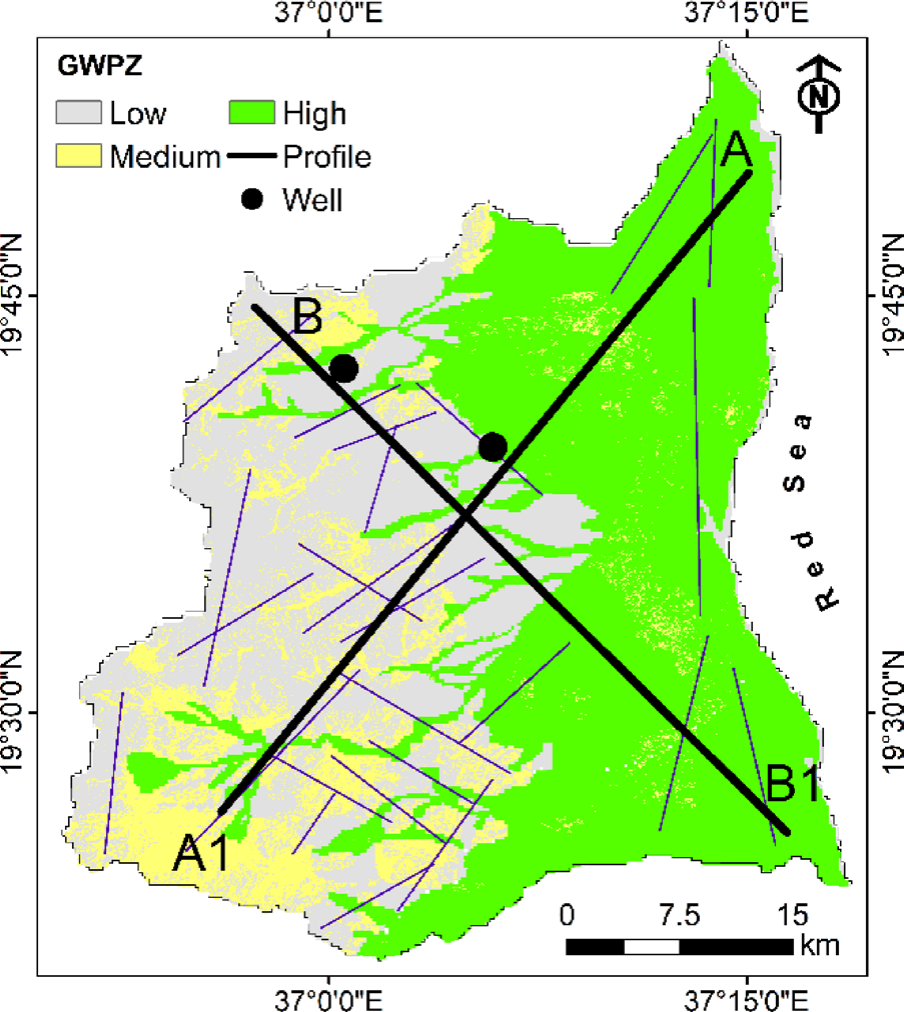
The groundwater potential zones obtained for integrated recharge influencing factors.

The integrated GWPZ map of Port Sudan presents a clear spatial delineation of groundwater prospectivity (Fig. 9). The CR is 0.1, indicating an acceptable level of consistency in the weighting process and minimizing potential subjectivity in assigning weights to the various parameters^66^. This final composite map reveals an east-west dichotomy in groundwater potential across the study area, with three distinct categories of potential zones: high, medium, and low. The high groundwater potential zones, predominantly occupy the eastern portion of the study area, extending from the northeastern coastal region southward in a broad swath that covers approximately 40–45% of the total area. This eastern zone of high potential coincides remarkably with the distribution of alluvial deposits^30^. The superior groundwater prospects in this region result from the optimal convergence of multiple favorable factors: permeable alluvial geology, gentle topographic slopes, moderate to high lineament density at depth, and adequate rainfall despite being relatively lower than western areas. These high potential zones extend westward along major drainage channels, creating finger-like projections into otherwise low potential areas, indicating the significant influence of both structural lineaments and alluvial-filled valleys as preferential pathways for groundwater movement and storage^67^.

The medium groundwater potential zones, form transitional belts primarily occurring at the interface between high and low potential regions. These zones cover approximately 10% of the study area and are most extensive in the southwestern and central portions. They typically occupy areas where moderate conditions exist across multiple parameters - such as areas with basement rocks exhibiting moderate fracturing, medium lineament density, and intermediate slopes^65^. The patchy, discontinuous nature of these medium potential zones highlights the complex spatial interplay between the various hydrogeological factors, where no single parameter dominates the groundwater potential classification. The low groundwater potential zones, predominantly occupy the western and northwestern portions of the study area, covering approximately 30–50% of the total area. These regions are characterized primarily by less permeable basement rocks, steeper slopes limiting infiltration^68^, and variable lineament density. Although these western areas receive relatively higher rainfall, the unfavorable geological conditions and topographic constraints substantially reduce effective recharge and storage capacity. Some low potential zones also appear as narrow fringes along the eastern coastal margin, representing areas where coral reef formations with limited freshwater potential predominate.

Several interpretative cautions must be considered when utilizing this GWPZ map for groundwater management decisions. For instance, the connectivity between fracture networks in low potential zones may be underestimated if they extend beneath alluvial cover in high potential areas, potentially creating hydraulic connections not evident from surface mapping. The temporal variability of certain parameters, particularly rainfall patterns and land use changes, introduces additional uncertainty, as this analysis represents conditions at a specific point in time rather than dynamic hydrological processes over seasonal or annual cycles. The sharp boundaries between potential zones should be interpreted as gradational transitions rather than abrupt hydrogeological changes, as groundwater systems rarely conform to such discrete spatial divisions. Areas classified as “low potential” may still contain localized productive aquifers, particularly at lineament intersections or in weathered basement pockets that may not be fully resolved at the current mapping scale. Conversely, zones identified as “high potential” may include localized areas of poor aquifer conditions due to facies variations, clay lenses. The coastal high potential zones specifically warrant cautious interpretation due to the risk of saltwater intrusion, which could significantly reduce freshwater availability despite favorable hydrogeological conditions.

### Validation of GWPZ through 2D gravity inversion

**Fig. 10:**
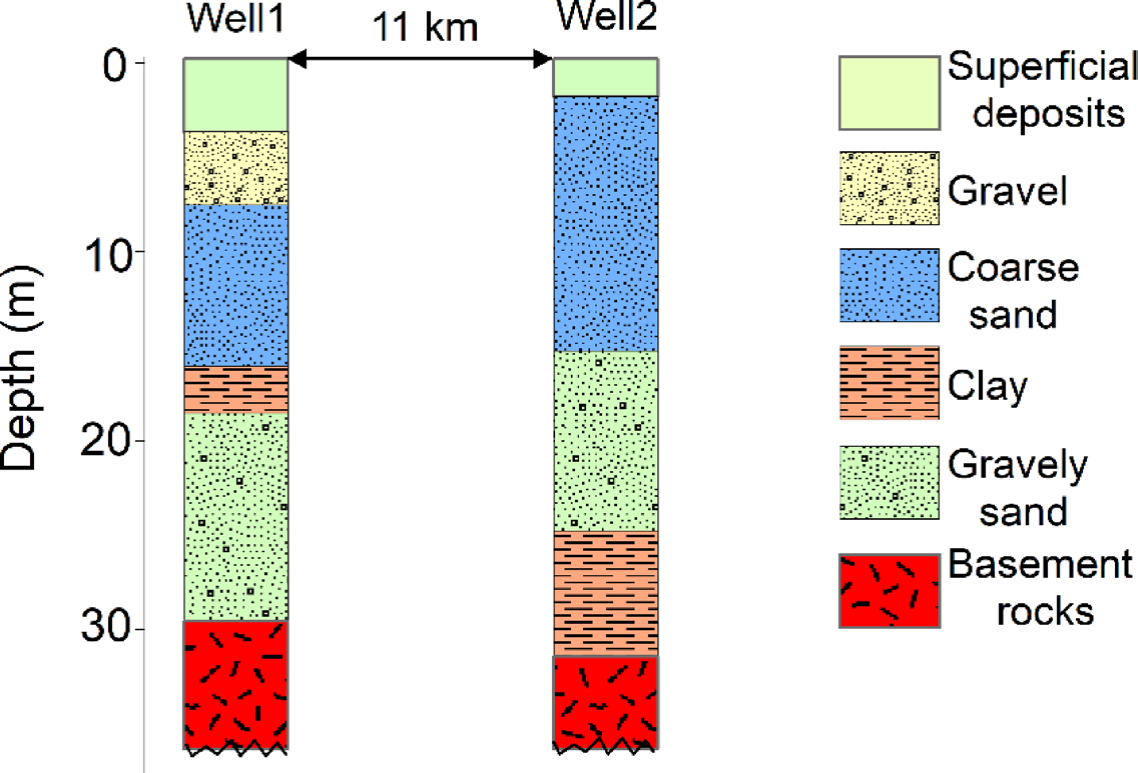
Lithological description along two wells used for constraining the gravity inversion.

This final GWPZ map provided guidance for groundwater development but should be considered a preliminary screening tool. Field verification remains essential before major development decisions^69^. In this study, gravity data inversion analysis along profiles is used to validate the AHP-derived GWPZ map by evaluating the distribution and depth of alluvial sediments as a main source of groundwater water. To minimize the inherent ambiguity of gravity inversion and enhance the geological reliability of the models, borehole data were integrated as constraints (Fig. 10). The borehole indicated distinct subsurface layering, with an upper sequence of unconsolidated alluvial deposits of approximately 29–33 m thickness overlying crystalline basement rocks. The gravelly sand and coarse sand units within the alluvial sediment sequence are saturated and constitute the main shallow aquifer units in the Port Sudan area. These coarse-grained sedimentary layers possess high porosity and permeability characteristics that enable significant groundwater storage capacity and efficient hydraulic transmission^37^. These lithological observations were crucial in constructing the initial two-layered model for gravity inversion. The upper layer was defined as low-density alluvial sediments (2.2 g/cm^3^), while the lower layer represented the higher-density basement complex (2.6 g/cm^3^). Based on the borehole data, the initial depth to the basement rock was uniformly set at 30 m along the profiles as a first approximation.

**Fig. 11:**
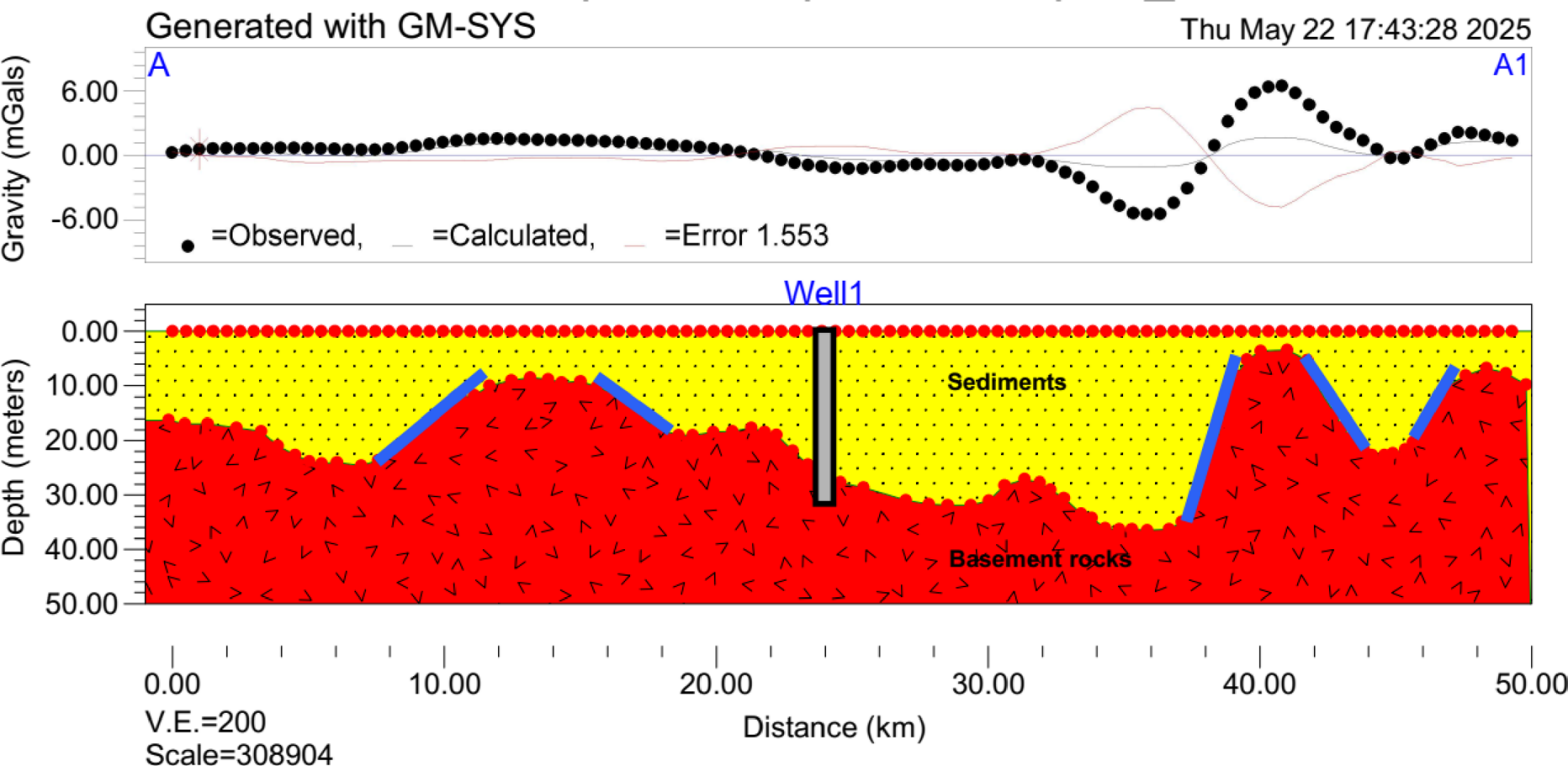
The 2D inversion of gravity data along Profile 1 showing the distribution of lithological and structural features (blue lines) with vertical exaggeration (V.E.) of 200.

Profile 1 (A–A1) (Fig. 11), which extends approximately 50 km in a northeast to southwest direction across the study area. The error analysis of the gravity inversion in this profile indicated that the correlation between observed and calculated gravity values is generally acceptable (RMS = 1.6 mGal); however, discrepancies are notable in areas with very high and very low gravity responses. These deviations suggest localized complexities or heterogeneities not fully captured by the two-layer model. As seen in the profiles, the misfit is most pronounced near fault zones and at the edges of thick alluvial basins, where the gravity signal exhibits sharp gradients. Generally, the profile revealed significant lateral variations in alluvial sediment thickness, primarily influenced by faulting and differential sedimentation. In the northern segment of the area (profile), the subsurface is characterized by a relatively uniform alluvial cover with an average thickness of about 20 m, overlying the crystalline basement.

A major structural transition is observed in the central portion of the profile, marked by a network of fault lines that produce abrupt vertical displacements in the basement surface. These faults define the boundaries of a fault-bounded local graben structure, where the basement drops sharply, creating substantial accommodation space for sediment infill. This structurally controlled basin is closely associated with high groundwater potential zones, as indicated by the AHP-derived classification, due to both increased sediment thickness and potential vertical connectivity between aquifer layers facilitated by fault-induced fracturing. In contrast, the southern part of the area exhibits a thinner accumulation of alluvial deposits, with maximum depths reaching only around 5 m. Although a few smaller grabens are observed in this region, they are relatively narrow and shallow. These conditions correspond to groundwater potential zones, reflecting the limited storage capacity and likely reduced hydraulic connectivity.

**Fig. 12:**
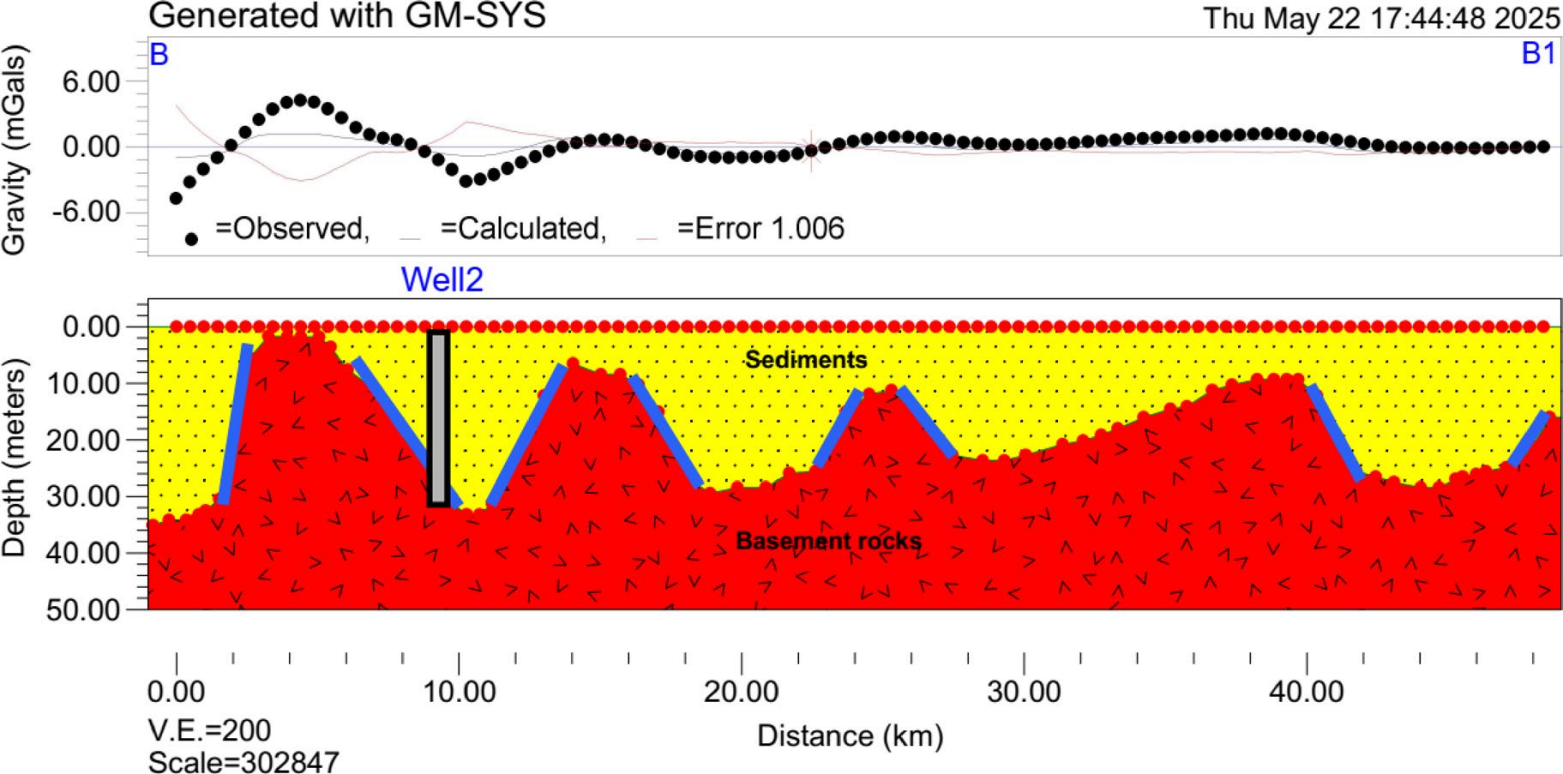
The 2D inversion of gravity data along Profile 2 showing the distribution of lithological and structural features (blue lines) with vertical exaggeration (V.E.) of 200.

Profile B–B1 (Fig. 12), which stretches approximately 48 km in a northwest to southeast direction across the study area. The error in this profile is lower compared to Profile 1, indicating an improved overall fit between the observed and calculated gravity responses (RMS = 1 mGal). However, the issue of low correlation persists at gravity peaks both high and low where sharp anomalies are present. This profile exhibits similar but more pronounced subsurface variations shaped by a complex fault network. The northwestern portion of the study area exhibits sedimentary cover ranging from 0 to 35 m, with thicker accumulations occurring only in localized depressions. The basement surface appears relatively elevated throughout most of this northwestern segment, reflecting the crystalline nature of the underlying rocks and their inherently low groundwater retention capacity. Although isolated pockets of greater sediment thickness exist, the predominantly shallow cover and elevated basement topography are consistent with the low-potential zone classification assigned to this region in the GWPZ map. In the middle parts of the area, the profile revealed multiple closely spaced faults that cause rapid downward offset of the basement, forming a structurally controlled transition zone. The southeastern parts displayed extensive alluvial sequences, with thicknesses exceeding 25 m in several locations. This thick fill occupies a fault-bounded grabens and is associated with high groundwater potential zones due to the enhanced accommodation space and increased porosity. The structural complexity and thickness variation within this segment reinforce the interpretation of the eastern part as a highly productive groundwater aquifer, directly supporting the AHP-based classification.

The gravity inversion results provided compelling validation for the AHP-derived groundwater zonation. The high groundwater potential zones in the eastern parts of the study area correspond to regions with maximum sediment thickness, where alluvial grabens offer significant pore space for groundwater storage^30^. These conditions are ideal for the development of productive aquifers, likely to include both shallow unconfined systems and deeper confined to semi-confined units where interbedded clay layers exist. In contrast, the low potential zones in the western basement-dominated areas exhibit minimal sediment cover and limited primary porosity. Groundwater in these regions is restricted to fractured basement rock, and its yield is expected to be more variable^70^. However, incorporating a more complex model such as a three-layer structure or horizontal density could potentially offer a more realistic representation of the subsurface. Doing so requires additional constraints from boreholes, seismic, or resistivity data to avoid overfitting. The localized misfits in the 2D model highlight zones of structural and lithological complexity where groundwater behavior may deviate from regional trends. As such, they mark areas where more detailed, targeted investigations are warranted. While the overall groundwater potential zonation remains robust, these anomalies suggest that in specific areas, particularly near faults or thick alluvial fills, the two-layer model has limitations and should be complemented by higher-resolution geophysical data such as seismic or electrical resistivity surveys for improved accuracy.

This study primarily focused on the shallow alluvial aquifer systems that represent the most accessible and productive groundwater resources in the Port Sudan area. However, it is important to acknowledge that weathered and fractured basement rocks can also serve as potential aquifers, particularly in areas where secondary porosity has been enhanced through tectonic fracturing and weathering processes. The investigation of these basement aquifer systems requires more comprehensive geological and structural studies, including detailed fracture network analysis, rock mass characterization, and assessment of weathering profiles. Moreover, the incorporation of groundwater level measurements alongside lithological and geophysical data will be essential to enhance the robustness of groundwater potential mapping. Integrating water table data can help validate and calibrate geophysical interpretations, particularly the thickness and extent of alluvial deposits inferred from gravity inversion. This combined approach will enable more accurate delineation of saturated zones, improve the understanding of groundwater flow dynamics, and strengthen the predictive reliability of the multi-criteria decision framework.

## Conclusions

This study employed an integrated approach combining lineament extraction, potential field analysis, multi-criteria decision analysis, and geophysical validation to assess groundwater potential in Port Sudan, Sudan. The lineament extraction analysis utilizing FVD, HG, TAD, and TAHG revealed a dominant structural framework characterized by NE-SW and NW-SE trending. These conjugate fracture systems, associated with Red Sea rifting processes. The AHP integration of six hydrogeological parameters - geology (37.1%), lineament density (17.6%), rainfall (24%), drainage density (9.6%), slope (7.6%), and land use/land cover (4%) - yielded a groundwater potential zonation map. The resulting GWPZ map delineated three potential categories: high potential zones predominantly in the eastern alluvial plains, low potential zones in the western basement terrain, and transitional medium potential zones at the interface between these geological domains. Gravity inversion validation along two representative profiles (A-A1 and B-B1) provided compelling subsurface verification of the AHP-derived groundwater potential zones. The inversion results, constrained by available borehole data, revealed substantial variations in alluvial thickness from minimal cover in western low-potential areas to exceptional accumulations in eastern high-potential zones.

While this research focused primarily on shallow alluvial aquifer systems, it acknowledges that weathered and fractured basement rocks may serve as alternative groundwater sources requiring specialized investigation through detailed geological, structural, and geophysical studies including seismic, electrical and logging techniques. The areas with limited sedimentary cover may contain localized groundwater resources in basement fracture networks. Moreover, this study is limited by the absence of seasonal groundwater fluctuations and water quality parameters, which are crucial for understanding long-term sustainability and potability. Future studies should incorporate these factors to provide a more comprehensive groundwater resource evaluation. The integrated methodology developed in this study provides a replicable framework for groundwater assessment in similar geological settings, demonstrating how remote sensing, geophysical, and GIS-based approaches can be effectively combined to guide sustainable water resource management. The research offers practical guidance for groundwater exploration prioritization, well siting optimization, essential for addressing water scarcity challenges in Port Sudan and comparable semi-arid coastal regions.

## Data Availability

The data that supports the findings of this study are available from the corresponding author upon reasonable request.
